# A Vibration Sensor-Based Method for Generating the Precise Rotor Orbit Shape with General Notch Filter Method for New Rotor Seal Design Testing and Diagnostics

**DOI:** 10.3390/s21155249

**Published:** 2021-08-03

**Authors:** Karel Kalista, Jindrich Liska, Jan Jakl

**Affiliations:** NTIS—New Technologies for the Information Society, Faculty of Applied Sciences, The University of West Bohemia, 30100 Pilsen, Czech Republic; jinliska@kky.zcu.cz (J.L.); jjakl@ntis.zcu.cz (J.J.)

**Keywords:** seal, magnetic bearings, general notch filter, harmonic excitation, rotor whirl

## Abstract

Verification of the behaviour of new designs of rotor seals is a crucial phase necessary for their use in rotary machines. Therefore, experimental equipment for the verification of properties that have an effect on rotor dynamics is being developed in the test laboratories of the manufacturers of these components all over the world. In order to be able to compare the analytically derived and experimentally identified values of the seal parameters, specific requirements for the rotor vibration pattern during experiments are usually set. The rotor vibration signal must contain the specified dominant components, while the others, usually caused by unbalance, must be attenuated. Technological advances have made it possible to use magnetic bearings in test equipment to support the rotor and as a rotor vibration exciter. Active magnetic bearings allow control of the vibrations of the rotor and generate the desired shape of the rotor orbit. This article presents a solution developed for a real test rig equipped with active magnetic bearings and rotor vibration sensors, which is to be used for testing a new design of rotor seals. Generating the exact shape of the orbit is challenging. The exact shape of the rotor orbit is necessary to compare the experimentally and numerically identified properties of the seal. The generalized notch filter method is used to compensate for the undesired harmonic vibrations. In addition, a novel modified generalized notch filter is introduced, which is used for harmonic vibration generation. The excitation of harmonic vibration of the rotor in an AMB system is generally done by injecting the harmonic current into the control loop of each AMB axis. The motion of the rotor in the AMB axis is coupled, therefore adjustment of the amplitudes and phases of the injected signals may be tedious. The novel general notch filter algorithm achieves the desired harmonic vibration of the rotor automatically. At first, the general notch filter algorithm is simulated and the functionality is confirmed. Finally, an experimental test device with an active magnetic bearing is used for verification of the algorithm. The measured data are presented to demonstrate that this approach can be used for precise rotor orbit shape generation by active magnetic bearings.

## 1. Introduction

Under certain circumstances, rotor vibrations in a steam turbine can cause rotor instability. Therefore, it is necessary to have a detailed knowledge of the individual components and assess their influence on the stability of the steam turbine. One of the components contained in the detailed examination is labyrinth seals. The primary purpose of seals is to minimize steam leakage between stages with different pressure levels. However, it was found that with increasing nominal turbine power, seals also significantly affect the rotor stability similarly to bearings and cannot be neglected. The rotor vibration and steam flow in the varying clearance between rotor and seal exert additional reaction forces acting on the whirling rotor which may increase its vibration and lead to machine instability. The seal is usually modelled as a two-dimensional system with dynamic stiffness and damping coefficients [1]. These coefficients are then substituted to the overall turbo-generator matrix model and the system stability can be assessed. The seal dynamic coefficients are calculated by CFD (computational fluid dynamics) software during its design.

However, results from numeric algorithms for turbulent flow within the seal are not consistent. In [2], the author presents a survey of seal coefficients from various sources and confirms that significant differences exist in the prediction of dynamic force coefficients for gas labyrinth seals. Therefore, experimental identification is necessary to validate the seal models. However, experimental measurements are almost impossible to carry out on real steam turbines. Therefore, specialized experimental test rigs need to be developed. The experimental tests rigs are considerably smaller than the real turbines and allow adjustment of the required operational condition and measurement of the rotor vibration and fluid-induced forces acting on the rotor within the seal. Measured data are then processed in the time or frequency domain and the seal dynamic coefficients are identified [3].

Construction of such a test device is quite challenging because it is necessary to excite and measure rotor vibration in the order of tens of micrometre and measure the fluid-induced forces in the order of few Newtons. Especially minimizing the force measurement uncertainties is challenging. Experimental testing of the seal rotordynamic force coefficients started decades ago. One of the first experiments with centred circular whirl orbit controlled by active feedback was described by Wright in 1983 [4]. This approach was adopted by Childs in 1986 [5] and used for small static eccentricity position of the rotor. The publication [6] brings a comprehensive review of experimental estimation of the rotor dynamic parameters of seals. Technological advances have made it possible to use a magnetic bearing in test equipment to support the rotor and as a contactless force exciter. The seal test rig with magnetic bearings was described by multiple publications, e.g., [7,8,9]. The advantage of active magnetic bearing (AMB) is the ability to control the vibrations of the rotor and to excite the desired shape of the rotor orbit (whirl).

The CFD methods usually presume a specific rotor orbit shape, usually circular. In order to be able to compare the experimental results with CFD simulations, the rotor orbit shape in AMB during the experiment must be as similar as possible to the simulation one. The rotor vibration signal must contain the specified dominant components, while the others must be attenuated. The main source of disturbances is mass unbalance of the rotor. Rotor unbalance occurs when the principal axis of inertia and the geometrical axis of the rotor are not coincident. This is due to manufacturing inaccuracies, material inhomogeneity or assembly tolerances and it can be caused also by the anisotropic magnetic bearing characteristics. The rotor field balancing method is usually applied; however, when the unbalance distribution is changing during operation, the residual unbalance always occurs. Unbalance causes undesirable synchronous rotor vibrations during operation which deteriorate the desired shape of the rotor orbit.

To attenuate synchronous unbalance vibration, the AMB position controller can be equipped with an active vibration compensator (AVC). The general principle of an AVC is to inject the synchronous compensation signal into the closed-loop control system of AMB to minimize the effect of the unbalance. In [10], the general notch filter algorithm (GNF) is described and used to eliminate the synchronous magnetic force to prevent AMB amplifier dynamic saturation. This unbalance compensation approach allows the rotor to spin around its inertia axis. Another connection of GNF into the closed loop allows the rotor to spin around its geometric axis. These two approaches are compared in [11] and other modifications of GNF can be found in, e.g., [12,13,14]. The advantage of GNF is that it does not require an AMB rotor system model and does not increase the AMB controller order significantly. In [10], a transformation matrix is used in the GNF circuit to tune the closed loop stability. In [11], the transformation matrix is replaced by a phase-shift angle which simplifies the stability setting.

On the other hand, the generation of precise rotor orbit shape is the opposite task to the aforementioned unbalance compensation. Usually, the harmonic signals with proper amplitude and phase are injected into the control loop of each AMB axis in order to excite the required harmonic rotor vibration. As the AMB rotor system parameters are not exactly the same for all axes, the amplitudes and phases of the injected signals in each axis are different. Moreover, the AMB axes are coupled and interact with each other. The amplitude and phase of these signals are also dependent on operational conditions. Therefore, adjusting the appropriate amplitudes and phases of these injected signals for each axis manually can be inconvenient.

In this article, a novel adaptive algorithm for the control of elliptic rotor orbit based on GNF is presented. The algorithm is explained and verified by simulations. Afterwards, the experimental test rig is described and the adaptive orbit generation algorithm is implemented and used in the real device. Significant rotor vibration was observed at the first (1X) and third (3X) multiples of a rotation speed of 3000 rpm during the previous experiments. To reach as precise a rotor orbit as possible, multiple GNF blocks in the AMB control loop are used simultaneously to compensate for the rotor synchronous vibration (1X, 3X). During the experimental identification of seals, asynchronous circular whirling of the rotor is presumed. The vibration amplitude must be high enough to induce measurable seal forces. The rotor circular orbit properties were assessed based on AMB performance limits. The seal identification experiments were carried out with rotor circular orbit with a radius of 50 µm and excitation frequency of 25 Hz. The rotor spin speed was 3000 rpm.

Firstly, the simulation of the rotor vibration and rotor orbit generation is presented. Afterwards, the implementation of the algorithm to the control loop of the real test rig is described. Lastly, the measured data and the performance of the algorithm is discussed.

## 2. Magnetic Bearing Control and Compensation Methods

This study deals with achieving a specified rotor orbit shape with the use of the AMB. In this section, the fundamental theory of AMB and the algorithm of GNF are explained. Finally, the novel orbit generation approach based on the GNF method is introduced.

### 2.1. AMB Rotor System

**Figure 1:**
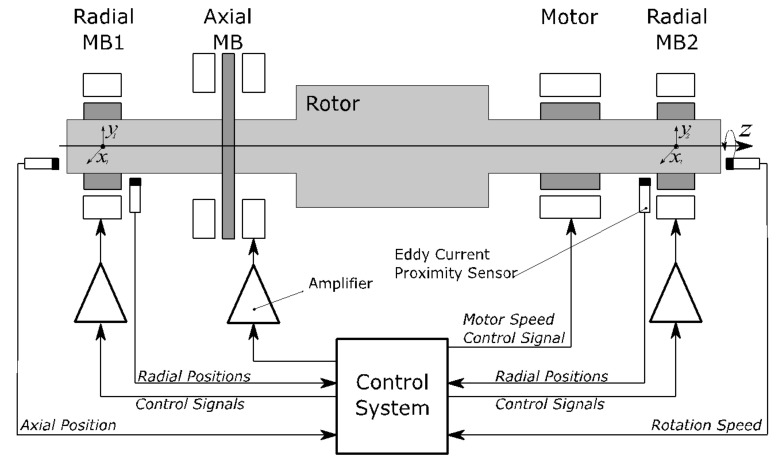
AMB rotor system.

A scheme of a fully suspended AMB system with a horizontal rotor levitating in active magnetic bearings is shown in Figure 1. The rotor is supported by two radial and one axial AMB which control five degrees of freedom of the rotor. The rotor spinning around the axial axis is driven by a motor and the rotation speed of the shaft is measured.

**Figure 2:**
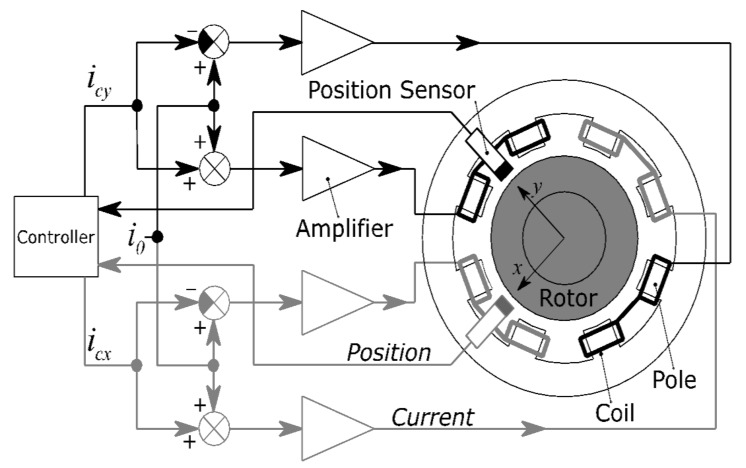
Radial magnetic bearing in differential drive mode.

A standard 8-pole radial active magnetic bearing and its connection are shown in Figure 2. The radial bearing contains four electromagnets arranged in two differential drives. The opposing electromagnets operating in the differential driving modes exert electromagnetic force on the rotor in two perpendicular axes. The rotor displacement from the centre position in each axis is measured by eddy-current sensors, which are located in the AMB housing. The radial AMB can thus control the position of the rotor in the bearing plane in two orthogonal axes, x and y. These axes are usually oriented at plus and minus 45 degrees from the vertical direction to distribute the gravitational force compensation uniformly between both differential drives. The rotor displacements are instantaneously measured and processed in a position controller which generates control signals for the amplifiers which adjust the electromagnet coil currents. The closed loop control is necessary to maintain the rotor levitation because the magnetic levitation is inherently unstable. The touchdown ball bearings support the rotor when it is not in levitation and thus prevent damage to the electromagnets. The AMBs have several advantages over conventional mechanical bearings such as no mechanical wear, low friction and absence of lubrication, low noise and high rotational speeds. On the other hand, magnetic bearings have much lower stiffness compared to mechanical bearings, so the rotation of the rotor is significantly affected by shaft unbalance. However, one of the advantages of AMB is the ability to adjust the mechanical properties during operation. The controller can be equipped with active vibration compensation to eliminate the undesired behaviour.

The resulting net force acting on the rotor for one differential driving mode is described by Equation (1) [15], where x denote the rotor displacement from a centered position, $s_{0}$ is the nominal air gap, $i_{0}$ is the bias current, $i_{c}$ is the control current and k is the construction constant.

(1) $$f_{m}=k\left(\frac{{\left(i_{0}+i_{c}\right)}^{2}}{{\left(s_{0}-x\right)}^{2}}-\frac{{\left(i_{0}-i_{c}\right)}^{2}}{{\left(s_{0}+x\right)}^{2}}\right)$$

To design the AMB controller based on linear system theory, the nonlinear Equation (1) is linearized at the operating point which is defined by bias current $i_{0}$ and nominal air gap$\;s_{0}$. The linearized expression of the electromagnetic force is obtained by the following equation:

(2) $$f_{m}={\left.\frac{\partial f_{m}}{\partial i_{c}}\right|}_{\begin{array}{l} i_{c}=0\\ x=0 \end{array}}\cdot i+{\left.\frac{\partial f_{m}}{\partial x}\right|}_{\begin{array}{l} i_{c}=0\\ x=0 \end{array}}\cdot x=k_{i}i_{c}+k_{s}x$$

where $k_{i}$ is the current stiffness and $k_{s}$ is the position stiffness of the AMB. These coefficients are specified by the AMB manufacturer.

A rotor supported by AMB is naturally a multi-input multi-output (MIMO) system. However, when the axis coupling is not significant, it can be neglected. It is usually the case for rotors with a low gyroscopic effect [15]. The system can be decoupled and controllers for each axis can be designed separately as a single-input single-output (SISO) system. According to Newton’s second law of motion $f=m\ddot{x}$ and Equation (2), the SISO transfer function of magnetic levitation is given by:

(3) $$F_{AMB}\left(s\right)=\frac{X\left(s\right)}{I\left(s\right)}=\frac{k_{i}}{ms^{2}-k_{s}}$$

where m is the corresponding amount of the rotor mass and s is the complex variable. The other parts of the AMB plant, such as amplifier and sensor, can be modelled as transfer functions of the first order filter according to Equation (4) and Equation (5), respectively, where $T_{a}$ and $T_{s}$ are the time constants of the power amplifier and sensor and $k_{a}$ a $k_{s}$ are the gains.

(4) $$\begin{array}{cc} F_{a}\left(s\right)=\frac{k_{a}}{T_{a}s+1}, & F_{s}\left(s\right)=\frac{k_{s}}{T_{s}s+1} \end{array}$$

The transfer function of the plant represented by Equation (5) includes AMB, sensor and amplifier.

(5) $$F_{p}\left(s\right)=\frac{k_{a}}{T_{a}s+1}\cdot \frac{k_{i}}{ms^{2}-k_{s}}\cdot \frac{k_{s}}{T_{s}s+1}$$

**Figure 3:**
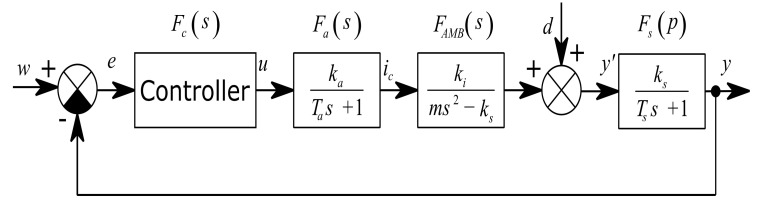
SISO model of AMB plant in closed loop control.

The PID controller is used in the AMB closed loop to control the rotor levitation. The PID is a widely used solution in industrial applications due to its good stability, reliability, undemanding implementation and convenient adjustment. The PID controller transfer function is described by the following equation:

(6) $$F_{c}\left(s\right)=P+\frac{I}{s}+\frac{Ds}{T_{f}s+1}$$

where P, I and D are the proportional, integration and differential gain coefficients. The coefficient $T_{f}$ is the derivative filtering time constant. The overall control loop is shown in Figure 3. The rotor unbalance is modelled as an additional signal *d* to the rotor position output. The unbalance signal is composed of one or more harmonic signals.

### 2.2. Unbalance Compensation

**Figure 4:**
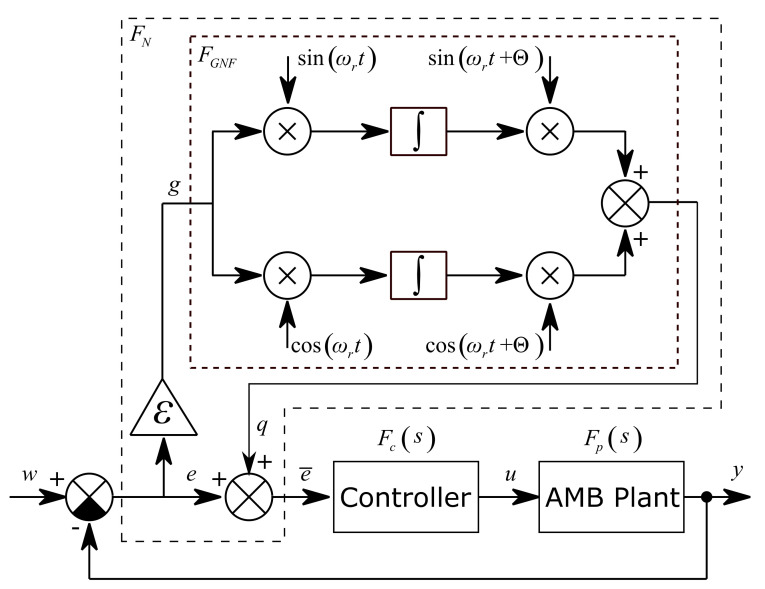
AMB closed loop control with the general notch filter as vibration compensator.

The unbalance compensation idea in this paper was adopted from [10], where the general notch filter (GNF) block is inserted into the closed loop to eliminate the synchronous coil current and make the rotor spin around its inertia axis. The general notch filter approach can be used to make the rotor spin around its geometric axis as is explained in [11]. In [11], the GNF with phase shift was used instead of the transformation matrix [10] to adjust the closed loop stability. In this paper, the GNF with phase shift is also used. The closed loop diagram of the unbalance compensation method is shown in Figure 4. The general notch filter$\;F_{N}$ consists of the general notch filter core $F_{GNF}$ which is inserted before the AMB position controller.

The rotor spin speed $\omega_{r}$ is constant or varying very slowly and it is continuously measured. Signals $sin\;\left(\omega_{r}t\right)\;$and $cos\;\left(\omega_{r}t\right)$ are generated in real time by an internal generator. The general notch filter core generates the corresponding harmonic signal to eliminate the synchronous unbalance vibration in the measured system output y. This can be called the two modulation step approach [11]. The position signal is multiplied by $sin\;\left(\omega_{r}t\right)\;$and $cos\;\left(\omega_{r}t\right)\;$to shift the frequency $\omega_{r}$ to zero. Then the signals are integrated and multiplied again by $sin\;\left(\omega_{r}t\right)\;$and $cos\;\left(\omega_{r}t\right)\;$to shift the frequency $\omega_{r}$ back. The integrations cause the elimination of the DC component, i.e., the shifted frequency$\;\omega_{r}$. The speed of convergence is adjusted by gain ε. If ε is equal to zero, the convergence is frozen, and the GNF behaves like a feed forward compensator. The convergence-speed parameter ε influences the closed loop stability and is usually adjusted empirically in range 0≪ε≪1, [10]. The closed loop stability is also adjusted by the phase shift parameter Θ.

The general notch filter core block $F_{N}$ has the input g and the output q. The relation between the input and the output is described by the following equation:

(7) $$q\left(t\right)=\left[\begin{array}{cc} \sin\left(\omega_{r}t+\Theta \right) & \cos\left(\omega_{r}t+\Theta \right) \end{array}\right]\cdot \int \left[\begin{array}{c} g\left(t\right)\cdot \sin\left(\omega_{r}t\right)\\ g\left(t\right)\cdot \cos\left(\omega_{r}t\right) \end{array}\right]dt$$

The first and second derivatives of the output are shown in the following relations:

(8) $$\dot{q}\left(t\right)=\omega_{r}\left[\begin{array}{cc} \sin\left(\omega_{r}t+\Theta \right) & \cos\left(\omega_{r}t+\Theta \right) \end{array}\right]\cdot \int \left[\begin{array}{c} g\left(t\right)\cdot \cos\left(\omega_{r}t\right)\\ -g\left(t\right)\cdot \sin\left(\omega_{r}t\right) \end{array}\right]dt+\cos\left(\Theta \right)\cdot g\left(t\right)$$

(9) $$\ddot{q}\left(t\right)=\cos\left(\Theta \right)\cdot \dot{g}\left(t\right)-\omega_{r}\sin\left(\Theta \right)\cdot g\left(t\right)-\omega_{r}^{2}\cdot q\left(t\right)$$

The transfer function of the general notch filter core is derived from Equation (8) and Equation (9) using the Laplace transform and is expressed by the following equation:

(10) $$F_{GNF}\left(s\right)=\frac{Q\left(s\right)}{G\left(s\right)}=\frac{s\cdot \cos\left(\Theta \right)-\omega_{r}\cdot \sin\left(\Theta \right)}{\omega_{r}^{2}+s^{2}}$$

The transfer function of the GNF (Equation (10)) inserted into the closed loop describes the relation between the input e and filtered output$\;\bar{e}$.

(11) $$F_{N}\left(s\right)=1+\varepsilon \cdot F_{GNF}\left(s\right)=\frac{\omega_{r}^{2}+s^{2}+\varepsilon \left(s\cdot \cos\left(\Theta \right)-\omega_{r}\cdot \sin\left(\Theta \right)\right)}{\omega_{r}^{2}+s^{2}}$$

The general notch filter transfer function can be expressed in the frequency domain by substituting s=jω:

(12) $$F_{N}\left(j\omega \right)\overset{s=j\omega \;}{=}\frac{\omega_{r}^{2}-\omega^{2}+\varepsilon \left(j\omega \cdot \cos\left(\Theta \right)-\omega_{r}\cdot \sin\left(\Theta \right)\right)}{\omega_{r}^{2}-\omega^{2}}$$

The influence of the unbalance d on the system measured position output y can be described in the frequency domain as follows:

(13) $$F_{d}\left(s\right)=\frac{Y\left(s\right)}{D\left(s\right)}\overset{w=0}{=}\frac{1}{1+F_{N}\left(s\right)F_{c}\left(s\right)F_{p}\left(s\right)}$$

When the convergence-speed parameter is in the range 0≪ε≪1 and the value of ω is close to the rotor spin speed$\;\omega_{r}$, the transfer function $F_{N}$ tends to infinity and the transfer function of disturbance $F_{d}$ tends to zero which means the synchronous disturbances are attenuated. When the values ω and$\;\omega_{r}$ are very different, the transfer function $F_{N}$ tends to one and the transfer function $F_{d}$ corresponds to the sensitivity function of the closed loop.

### 2.3. Orbit Generation

**Figure 5:**
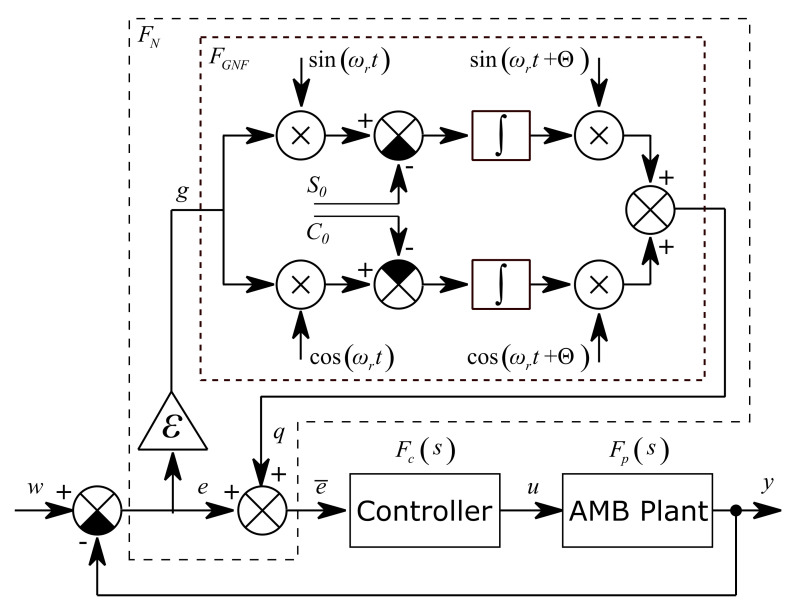
AMB closed loop control with the general notch filter as a vibration generator.

The precise harmonic vibration generation approach is based on the general notch filter as well as the unbalance compensation. The general notch filter core is modified as shown by Figure 5. The constant values $S_{0}$ and $C_{0}$ are subtracted from the product of the input signal with sine and cosine, respectively.

The constants $S_{0}$ and $C_{0}$ are derived from the required amplitude A and phase Φ of the output harmonic signal which can be described by a sinusoidal function, according to the following equation:

(14) $$y=A\sin(\Omega t+\Phi )=A\sin\left(\Phi \right)\cos(\Omega t)+A\cos\left(\Phi \right)\sin(\Omega t)$$

The coefficients $S_{0}$ and$\;C_{0}$, shown in Figure 5, correspond to the constants in Equation (14), which are scaled by ε/2, according to the following equation:

(15) $$\left[\begin{array}{c} S_{0}\\ C_{0} \end{array}\right]=\frac{\varepsilon }{2}\left[\begin{array}{c} A\cos\left(\Phi \right)\\ A\sin\left(\Phi \right) \end{array}\right]$$

**Figure 6:**
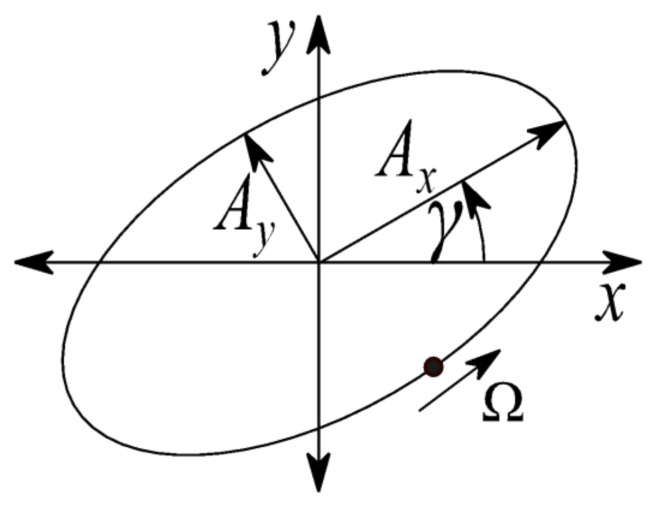
Elliptic orbit.

Figure 6 shows the general elliptic orbit in the Cartesian coordinate system$\;\left(x,y\right)$. The ellipse is described by two semi axes$\;A_{x}$, $A_{y}$ and the rotation angle γ.

When γ is equal to zero, the ellipse is expressed by the following equation:

(16) $$\left[\begin{array}{c} x\\ y \end{array}\right]=\left[\begin{array}{c} A_{x}\cos\left(\Omega t\right)\\ A_{y}\sin\left(\Omega t\right) \end{array}\right]$$

The application of rotation transformation is described as:

(17) $$\begin{array}{cc} \left[\begin{array}{c} x\\ y \end{array}\right] & =\left[\begin{array}{cc} \cos\left(\gamma \right) & -\sin\left(\gamma \right)\\ \sin\left(\gamma \right) & \cos\left(\gamma \right) \end{array}\right]\left[\begin{array}{c} A_{x}\cos\left(\Omega t\right)\\ A_{y}\sin\left(\Omega t\right) \end{array}\right]\\ & =\left[\begin{array}{c} A_{x}\cos\left(\gamma \right)\cos\left(\Omega t\right)-A_{y}\sin\left(\gamma \right)\sin\left(\Omega t\right)\\ A_{x}\sin\left(\gamma \right)\cos\left(\Omega t\right)+A_{y}\cos\left(\gamma \right)\sin\left(\Omega t\right) \end{array}\right] \end{array}$$

The coefficients $S_{0}$ and $C_{0}$ for generating required elliptic orbit by GNF are obtained from Equation (17) as:

(18) $$\left[\begin{array}{c} S_{x0}\\ C_{x0}\\ S_{y0}\\ C_{y0} \end{array}\right]=\frac{\varepsilon }{2}\cdot \left[\begin{array}{c} -A_{y}\cdot \sin\left(\gamma \right)\\ A_{x}\cdot \cos\left(\gamma \right)\\ A_{y}\cdot \cos\left(\gamma \right)\\ A_{x}\cdot \sin\left(\gamma \right) \end{array}\right]$$

The speed of convergence and stability of the algorithm can be adjusted by parameters ε and Θ.

## 3. Results

The aforementioned methods are verified by both simulations and experiments on the real AMB rotor system. The simulation model is an approximation of the real AMB rotor system. The exact system model is not available due to the complex design of the rotor, which is described in Section 3.2. Identification of the mathematical model of the system is beyond the scope of this paper and will be carried out in further work. The PID controller is used for its feasibility and low resource usage in an FPGA (field programmable gate array). In addition, the PID controller can be tuned even if the exact mathematical model of the system is not known.

### 3.1. Simulations

**Figure 7:**
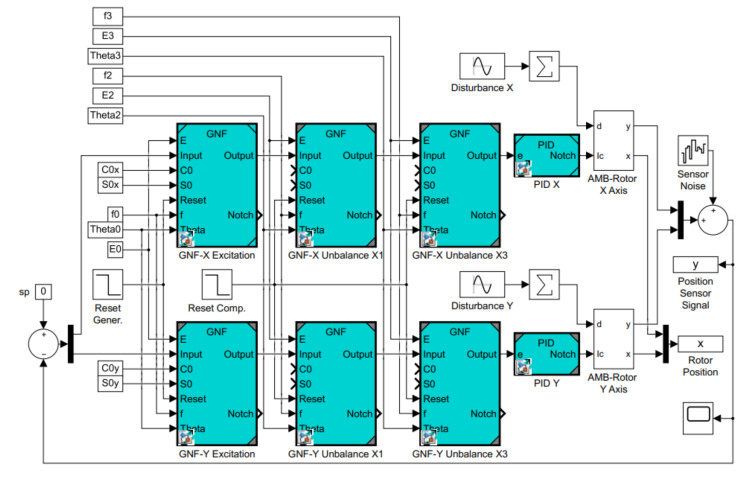
Simulation block scheme.

The Matlab/Simulink simulation block scheme of the decentralized control of one radial AMB using a PID controller is shown in Figure 7. The scheme is composed of two control loops for AMB axes x and y where each axis is extended with three GNF blocks. The AMB rotor model is implemented based on the introduced scheme in Figure 3. The model parameters in Table 1 correspond to the real AMB rotor system. The rotor model does not include flexible modes which are located far from the operating speed range 0–50 Hz.

The disturbance signal consists of the first (1X) and the third (3X) multiple of the rotor spin speed $\left(50\;\mathrm{Hz}\right)$ to simulate the disturbances that occurred in the real device during previous experiments. The sensor noise is simulated with the use of the block Band-Limited White Noise in Matlab. The simulation setting and parameters of the excited elliptic orbit are shown in Table 2.

**Figure 8:**
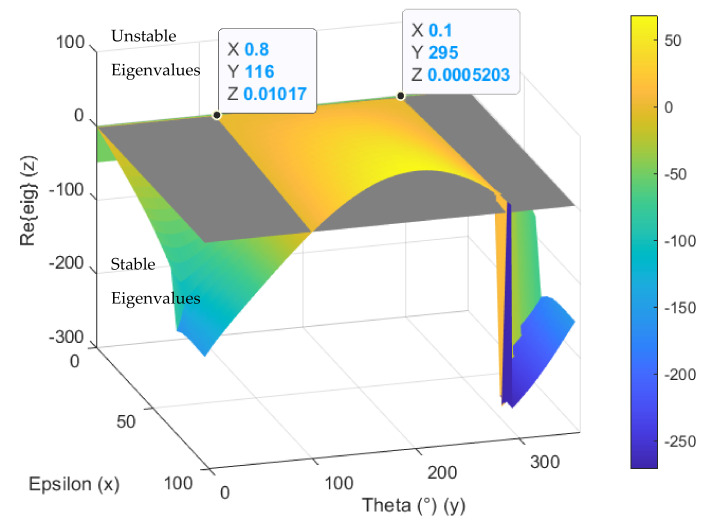
Critical eigenvalue dependence on parameters ε and Θ for unbalance frequency 50 Hz.

The stability of the closed loop system depends on parameters ε and Θ and can be evaluated by the eigenvalue analysis. The dependence of the real part of the critical eigenvalue on parameters ε and Θ for unbalance frequency 50 Hz is shown in Figure 8. The grey plane shows the boundary between stable eigenvalues with a negative real part and unstable eigenvalues with a positive real part. This confirms that the stability is mainly affected by parameter Θ [11]. The value of parameter Θ must be chosen in the stable region. Parameter ε is chosen small to get large bandwidth [11].

**Figure 9:**
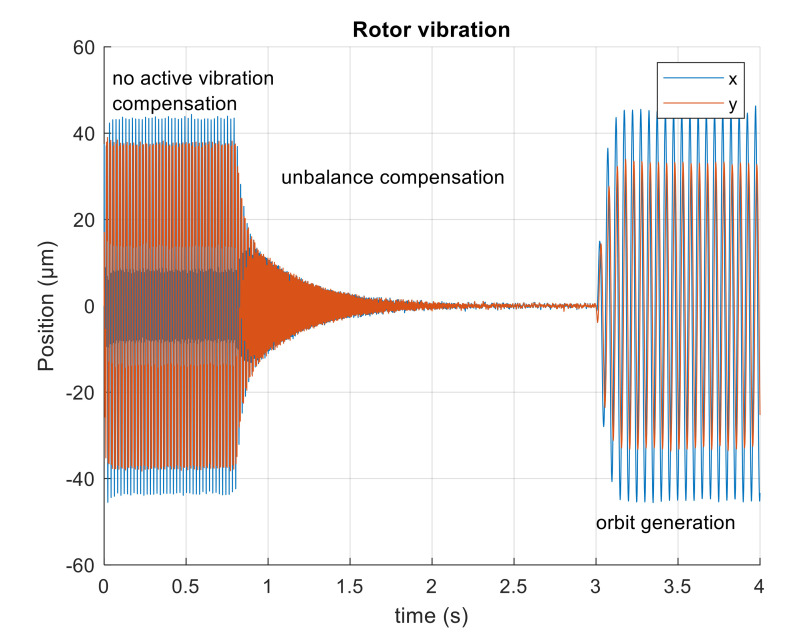
Simulated position of the rotor in axes, x and y.

**Figure 10:**
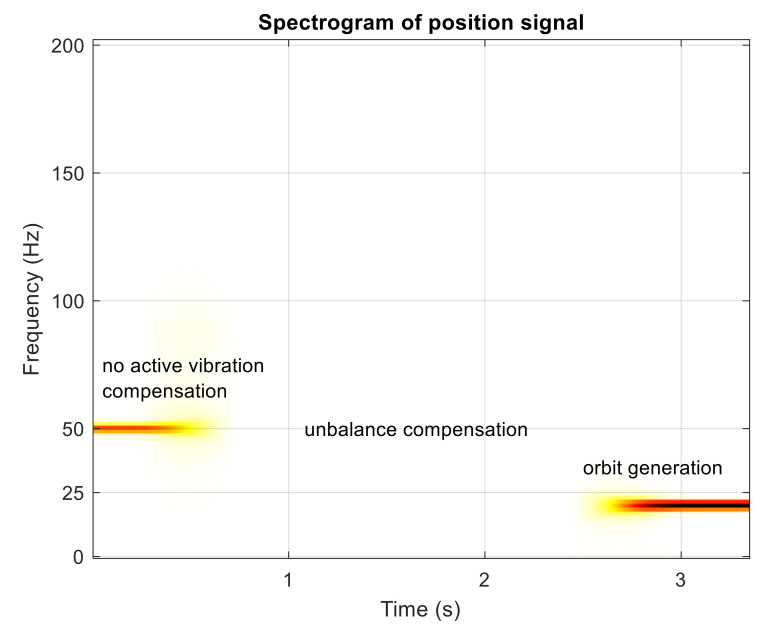
Spectrogram of simulated position signal in the x axis.

**Figure 11:**
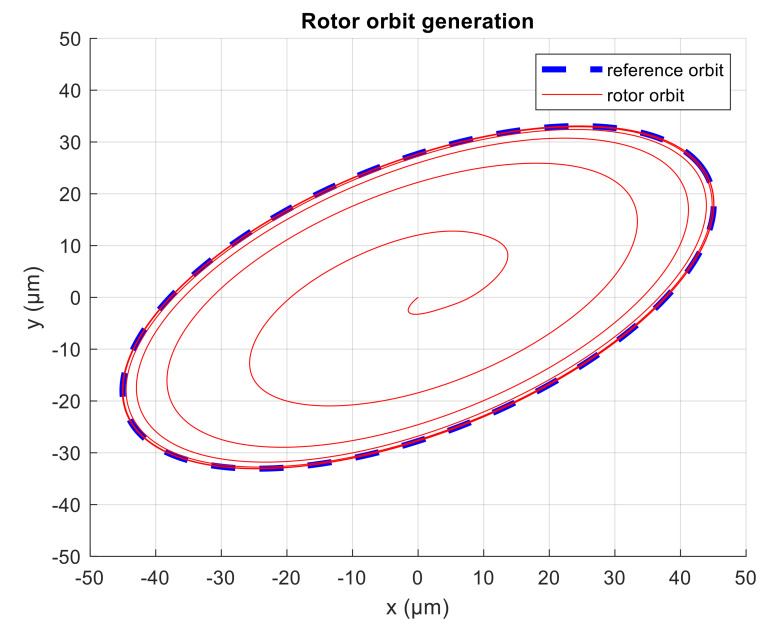
Rotor orbit during simulation.

The simulation experiment starts with no active vibration compensation and the output position signal containing the disturbance components (1X, 3X). Then, the unbalance compensation starts to reject the disturbances. Subsequently, the orbit generation starts and the required harmonic signals are excited to build the elliptic orbit which is specified by two semi-axes and rotation angles. The simulated position time signals in x and y axis are shown in Figure 9. The spectrogram of position signal in the x axis is depicted in Figure 10. The final progress of the orbit generation part is shown in Figure 11.

The simulations confirm the method validity and usage of multiple GNF blocks in a series for unbalance compensation and vibration excitation. When the parameters ε and Θ were chosen properly based on eigenvalue analysis, the algorithm attenuated the disturbances caused by unbalance and the desired rotor orbit shape was achieved.

### 3.2. Experimental Test Rig and Measurement

**Figure 12:**
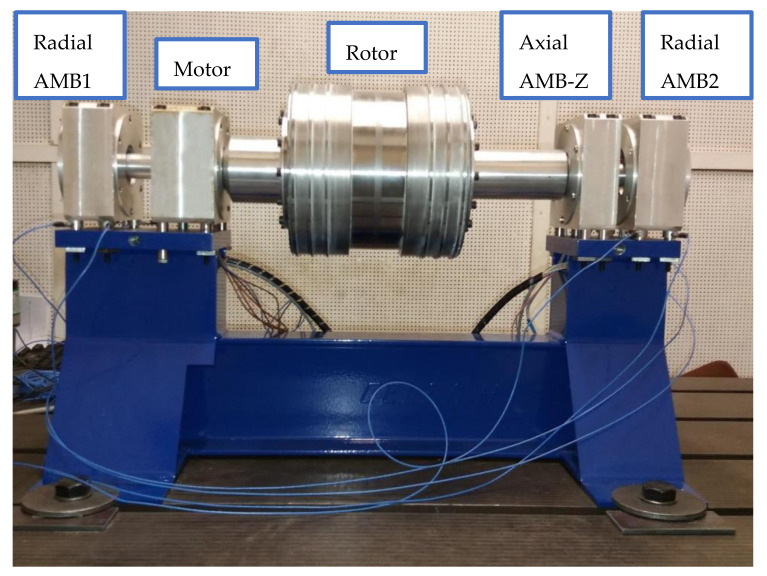
Experimental rotor supported by magnetic bearings.

The experimental test rig with active magnetic bearings is being built for the identification of seal dynamic force coefficients [3]. Figure 12 displays the test rotor supported with two radial active magnetic bearings and an axial bearing. The load of each magnetic bearing is 100 N. The rotor spinning around the axial axis is driven by a 3-phase 4-pole induction motor. The motor armature is press-fitted onto the shaft which passes through the motor housing. Therefore, no mechanical coupling is needed and the rotor can levitate without any contact with the stator. The operating speed range of the test rotor is 0−3000 rpm. The rotor phase mark signal is observed by the eddy-current sensor and the tachometer signal is converted to the rotation speed. The control system of the magnetic bearings is running on NI cRIO 9185 from National Instruments which is equipped with I/O cards. A decentralized PID controller is implemented in FPGA with the sampling frequency of 12500 Hz. The control algorithm includes multiple GNF for each AMB axis. The GNF are used for active vibration compensation and excitation of the desired rotor orbit. The physical parameters of the test rig correspond to the parameters used in the simulation, see Table 1. Both of the radial AMB housings are supported by four quartz load cells which measure the reaction force in a vertical direction between the housing and the base [3]. The weight of the hollow rotor is 8 kg and it consists of three parts, two aluminium flanges and a central steel test part. The first flexible mode of the rotor is about 200 Hz, which is above the operating speed range.

**Figure 13:**
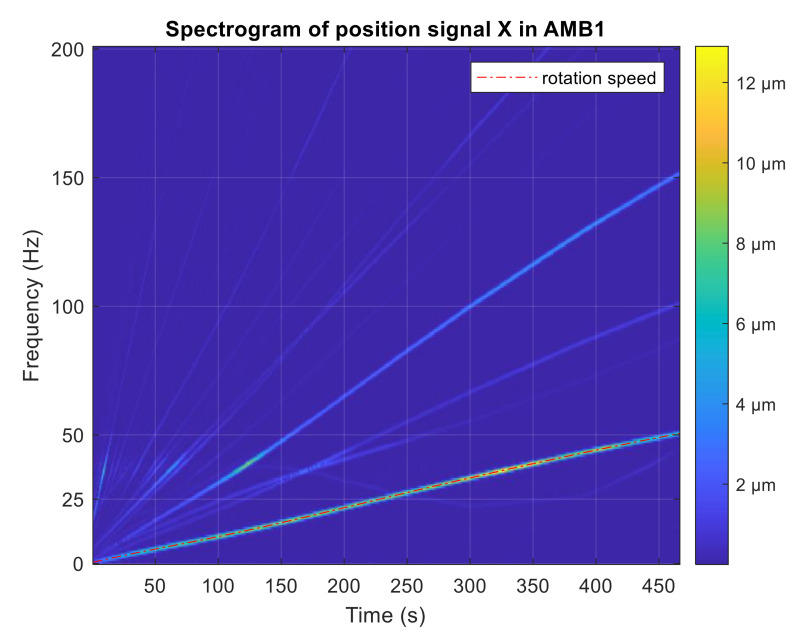
STFT spectrogram of position signal during run-up without AVC.

During the experiment, the levitation of the static rotor was stable using only the PID control of the AMB. The unbalance disturbances occurred when the rotor spin speed was increased. Field balancing with the use of trial weight was performed to balance the rotor. Figure 13 shows the STFT spectrogram of position signal in one AMB axis during run-up without AVC. The residual unbalance caused vibration of the rotor. The amplitude of vibration varies with speed up to approximately 12 µm.

**Figure 14:**
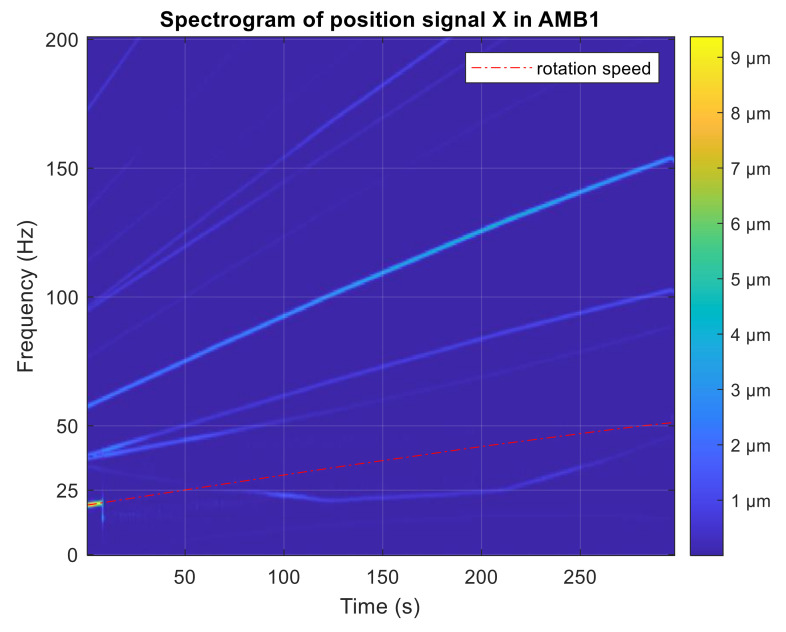
STFT spectrogram of position signal during run-up with AVC.

The active vibration compensation was started when the rotation speed reached 20 Hz and the unbalance was eliminated. The AVC algorithm becomes unstable as the spin speed was increased, therefore the phase shift parameter and the convergence-speed parameter were adjusted manually to ensure stable shaft rotation. Figure 14 shows the run-up test with a stable AVC setting.

**Figure 15:**
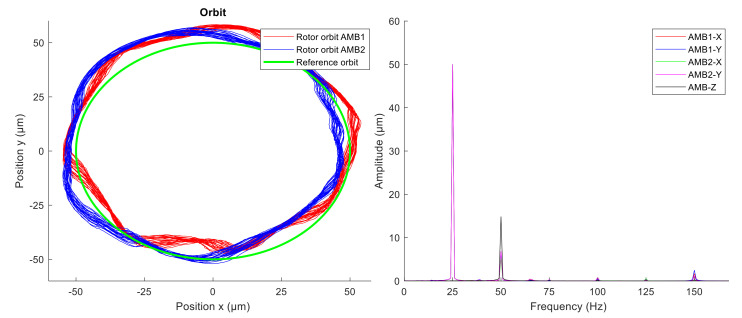
Measured rotor orbits (**left**) and signal amplitude spectra (**right**) without AVC.

The first orbit generation experiment was performed with constant rotor spin speed 3000 rpm without AVC. The generalized notch filter was used to excite the circular whirl motion of the rotor. The radius of the circular orbit was 50 µm and the whirling frequency of the rotor was 25 Hz. Rotor vibrations were measured in the AMB housing by the integrated eddy-current sensors in axis x and y. Figure 15 shows rotor orbits measured in planes of radial magnetic bearings and related amplitude spectra of the measured position signals.

**Figure 16:**
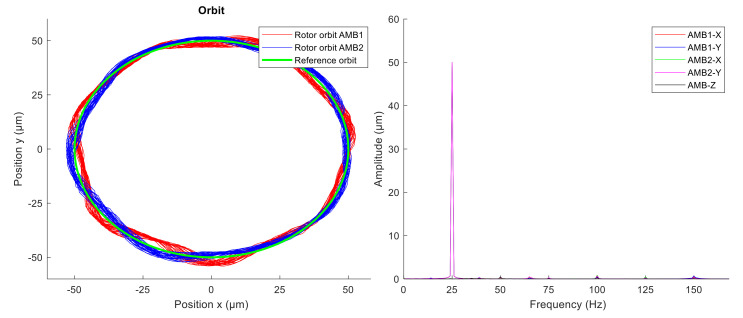
Measured rotor orbit (**left**) and signal amplitude spectra (**right**) with AVC.

The second experiment was performed with active vibration compensation. The GNF was used to compensate for the first and the third multiple of the rotor spin speed (1X, 3X). The measured rotor orbit and amplitude spectra are shown in Figure 16. The time interval of the displayed measured data is one second.

**Figure 17:**
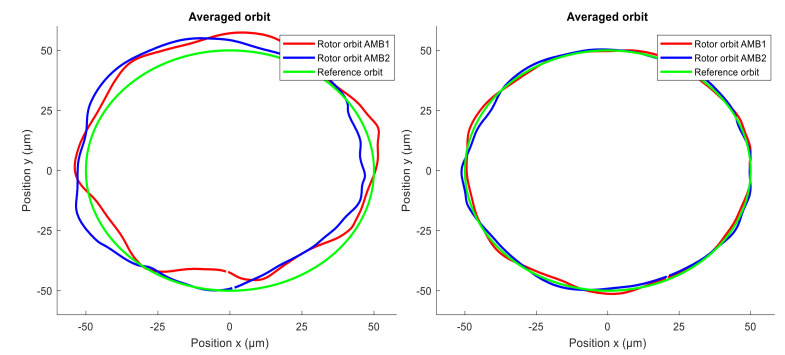
Average of measured rotor orbit: (**left**) without AVC; (**right**) with AVC.

At first, the unbalance compensation was verified during the run-up test from 0 to 3000 rpm. When the spin speed was 3000 rpm, the orbit generation was started. Desired circular orbit was generated and maintained by the novel modified GNF algorithm. The levitation of the spinning rotor which was whirling in circular orbit was stable. Comparing the amplitude spectra (Figure 15 and Figure 16), the vibration compensation by GNF eliminates the first and the third multiples (1X, 3X) of the rotor spin speed, which results in a more precise circular orbit shape. The measured position time signals in axes x and y were averaged to obtain a representative orbit shape, which was compared with a reference circle, as shown in Figure 17. The average error between the measured averaged orbit and the reference is listed in Table 3. The active vibration compensation improves the orbit shape by 81 and 85 percent in AMB1 and AMB2 respectively.

## 4. Discussion and Conclusions

The main idea of this paper is to show that the active vibration compensation algorithm for AMB rotor system based on a general notch filter can be successfully used for generating precise rotor orbit during rotordynamic experiments. A known GNF algorithm was used for the unbalance compensation and simultaneously the novel modified GNF was introduced and used to generate the required rotor orbit.

The experimental investigation of the rotordynamic characteristics of seals requires a precise shape of the rotor orbit. The rotor vibration signal must contain the specified dominant components while the others, usually caused by unbalance, must be attenuated. During previous rotordynamic experiments, a high amplitude of vibration was observed at 1X and 3X, resulting in an unsatisfactory shape of the rotor orbit. Therefore, multiple general notch filters in a series were used simultaneously to actively compensate for the unbalance vibrations. To excite the desired shape of the rotor orbit, corresponding harmonic signals were injected into each control loop of the AMB axis. During previous tests, the amplitude and phase of these signals were adjusted manually which was inconvenient and time consuming. Therefore, the novel modified GNF was used to excite the asynchronous rotor whirl. Using the correct parameters, the algorithm excites the desired shape automatically and maintains it.

The usage of multiple GNF was simulated with a simplified model of the AMB rotor system with physical parameters corresponding to the real device. The simulation results confirmed the functionality of the algorithm which was then implemented in FPGA together with the AMB controller. The stability of the vibration compensation part of the algorithm was adjusted during the experimental test to ensure stable rotor levitation covering the whole speed range 0–3000 rpm. Then, the orbit generation part was started to excite the rotor asynchronous vibration with a circular orbit with a radius of 50 µm. The algorithm generated the required orbit shape automatically within a few seconds depending on the settings and the magnetic levitation of the rotor remained stable. The generated orbits were compared in cases with and without active vibration compensation. The compensation improved the average error by 81 and 82 percent on AMB1 and AMB2, respectively.

One of the main benefits of the modified GNF is achieving the rotor orbit automatically compared to the injection of the required harmonic signal to each AMB axis separately. Although the axes of the AMB rotor MIMO system interact with each other, the algorithm adapts the excitation signal to converge to the desired rotor orbit. Finally, when the required orbit is reached, the adaptation can be stopped to eliminate the GNF algorithm which may affect the rotordynamic experiment in an undesirable way.

## Figures and Tables

**Table 1:** Parameters of the AMB rotor system and PID control.

Name	Value
Rotor mass $\left(m\right)$	8 kg
Current stiffness$\;\left(k_{i}\right)$	40 N/A
Position stiffness $\left(k_{x}\right)$	279,000 N/m
Power amplifier gain $\left(k_{a}\right)$	0.53 A/V
Power amplifier time constant $\left(T_{a}\right)$	175 µs
Position sensor gain $\left(k_{s}\right)$	40,000 V/m
Position sensor time constant $\left(T_{s}\right)$	250 µs
Bias current $\left(i_{0}\right)$	3 A
Maximal force	100 N
Control loop sampling rate	12,500 Hz
PID proportional gain $\left(P\right)$	1.05
PID integral gain $\left(I\right)$	31.66
PID derivative grain $\left(D\right)$	0.0018
PID derivative filter time constant $\left(T_{f}\right)$	$7.3\times 10^{-5}$

**Table 2:** The simulation parameters.

Parameter	Value
Disturbance frequencies $\;\left(f_{1},\;f_{3}\right)$	50 Hz, 150 Hz
Disturbance amplitudes	20 µm, 10 µm
Noise power	1 × 10^−7^
Excitation frequency $\left(f_{0}\right)$	20 Hz
Ellipse semi-axis	50 µm, 25 µm
Ellipse rotation angle $\left(\gamma \right)$	30°
Theta 0,1,3 (Θ)	20°
E 0,1,3 (ε)	0.002

**Table 3:** Comparing of generated orbit shape.

Name	Average Error (µm)		Improvement (%)	
	AMB1	AMB2	AMB1	AMB2
Experiment without AVC	3.79	3.87	-	-
Experiment with AVC	1.35	1.03	81	85
